# A case report of autosomal recessive polycystic kidney disease with noncompaction of ventricular myocardium: coincidence or different manifestations of ciliopathy?

**DOI:** 10.1186/s12882-024-03642-7

**Published:** 2024-06-25

**Authors:** Weiran Zhou, Qingxia Du, Qinghua Liu, Xiaofang Liu, Lei Li, Hongxia Zhang

**Affiliations:** 1 Department of Pediatric Nephrology and Rheumatism and Immunology, Children‘s Hospital Affiliated to Shandong University, Jinan, Shandong Province China; 2 Department of Pediatric Nephrology and Rheumatism and Immunology, Jinan Children’s Hospital, Jinan, Shandong Province China; 3 Department of Cardiovascular Medicine, Children’s Hospital Affiliated to Shandong University, Jinan, Shandong Province China; 4 Department of Ultrasonography, Children’s Hospital Affiliated to Shandong University, Jinan, Shandong Province China

**Keywords:** Autosomal recessive polycystic kidney disease, Noncompaction of ventricular myocardium, Heart failure, Children, Case report

## Abstract

**Background:**

Autosomal recessive polycystic kidney disease (ARPKD) is a rare inherited cystic disease characterized by bilateral renal cyst formation and congenital liver fibrosis. Cardiovascular disorders such as noncompaction of ventricular myocardium (NVM) have not been reported with ARPKD.

**Case presentation:**

A 5-month-old girl was examined after presenting with a fever and turbid urine for one day and was diagnosed as urinary tract infection. Urinary ultrasound showed multiple round, small cysts varying in size in both kidneys. Genetic testing revealed two heterozygous mutations and one exon deletion in the polycystic kidney and hepatic disease 1 gene, indicating a diagnosis of ARPKD. During hospitalization, she was found to have chronic heart failure after respiratory tract infection, with an ejection fraction of 29% and fraction shortening of 13%. When the patient was 15 months old, it was found that she had prominent trabeculations and deep intertrabecular recesses with the appearance of blood flow from the ventricular cavity into the intertrabecular recesses by echocardiography. The noncompaction myocardium was 0.716 cm and compaction myocardium was 0.221 cm (N/C = 3.27), indicating a diagnosis of NVM. Liver and kidney function remained normal during four-year follow-up.

**Conclusions:**

This is the first report of NVM in a patient with ARPKD. It is unsure if the coexistence of NVM and ARPKD is a coincidence or they are different manifestations of ciliary dysfunction in the heart and kidneys.

**Supplementary Information:**

The online version contains supplementary material available at 10.1186/s12882-024-03642-7.

## Introduction

Autosomal recessive polycystic kidney disease (ARPKD) is an autosomal recessive inherited ciliopathy characterized by bilateral renal cysts and congenital liver fibrosis [1–3]. Its incidence rate is approximately 1/2000 and can lead to end-stage renal disease (ESRD) in approxImately 15–30% childhood/early adolescent patients [1, 3, 4]. Respiratory distress caused by pulmonary hypoplasia is the cause of death in 30 -50% of newborns with ARPKD [1, 2].

The primary pathogenic gene of ARPKD is *PKHD1* gene on chromosome 6 p21. It encodes fibrocystin/polyductin (FPC) -ciliated protein which was mainly distributed in the kidneys, liver, and pancreas [2]. Genetic testing can be particularly important in the diagnostic evaluation of patients with early-onset bilateral renal cystic disease and hepatic fibrosis with or without renal cysts in older patients. ARPKD patients may have extrarenal and extrahepatic manifestations including intracranial aneurysms, left ventricular hypertrophy, neurological abnormalities, abnormal ocular fundus, abdominal pain and deformities of the spine and limbs which can occur in patients of various ages and disease states [5].

In the cardiovascular system manifestation of ARPKD, systemic hypertension can cause left ventricular hypertrophy and congestive heart failure [5]; Noncompaction of ventricular myocardium(NVM) is a rare cardiomyopathy characterized by altered myocardial wall with prominent trabeculae and deep intertrabecular recesses, with an incidence of 0.014–1.3% among patients undergoing echocardiography. It could be diagnosed by echocardiography, magnetic resonance or multidetector computed tomography. The ratio of non-compacted layers to the compacted layers ≥ 2 at end diastole was considered for NVM. The prognosis varies upon the patient’s age, genetic predisposition, and cardiac function [6]. Here, we report a case of ARPKD with NVM and chronic heart failure. To our knowledge, this is the first report of ARPKD with NVM.

## Case report

A five-month-old girl experiencing a fever and turbid urine for one day was admitted to the hospital.The blood pressure was 89/53mmHg. Urinary examination revealed a white blood cell count of 10,085 /µl and 1815 /High power field, which led to a diagnosis of a urinary tract infection. The creatine was 19 µmol/L and the estimated glumorular filter rate was 121 ml/min/1.73m^2^ calculated by Schwartz formula. Urinary ultrasound measured the right kidney at 8.7 × 4.5 cm and the left at 8.2 × 4.4 cm which is enlarged to the child’s length 63 cm. Both kidneys had multiple round, small cysts varying in size. The parenchyma was hyperechoic. Liver ultrasonography indicated normal size and local calcification of the bile duct wall. There was no family history of any diseases. Prenatal ultrasound indicates mild dilation of the left renal pelvis with a width of approximately 0.8 cm at 38 weeks of pregnancy. Urine culture indicated that she was infected by Enterobacter cloacae which were multiple resistant and sensitive to meropenem. After the treatment with meropenem, the body temperature returned to normal and the white blood cells in urine decreased. Unfortunately, the child developed respiratory tract infections during hospitalization, presented with a mild cough, a body temperature of 38℃, irritability and crying, shortness of breath, and poor feeding. Physical examination revealed a heart rate of 170–180 beats per minute, respiration of 60–70 beats per minute, and liver enlargement. Electrocardiogram showed left ventricular high voltage and ST-T changes. Cardiac ultrasound showed enlarged left ventricular (2.8 cm) and atrial (1.2 cm) diastole diameters, with an ejection fraction (EF) of 29% and fraction shortening (FS) of 13%. She was diagnosed with heart failure and treated with furosemide, deacetylated anthocyanins, and milrinone. Blood pressure was 90/60 mmHg. Although the vital signs normalized after treatments, the patient still had low EF (41%) and FS (19%).

**Fig. 1:**
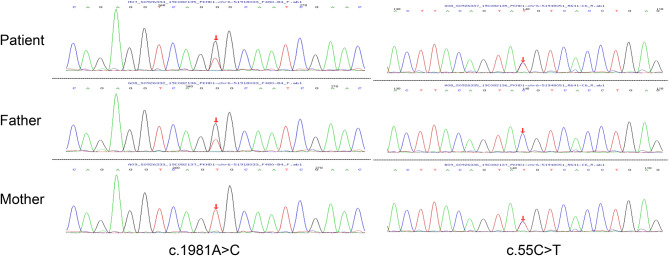
Whole exome sequencing revealed two missense mutations in the polycystic kidney and hepatic disease 1 (*PKHD1*) gene. c.1981 A > C (p.T661P) was inherited from her father, and c.55 C > T (p.R19C) was inherited from her mother

Whole exome sequencing revealed two missense mutations in the polycystic kidney and hepatic disease 1 (*PKHD1*) gene: c.1981 A > C (p.T661P) was inherited from her father(PM2 + PM3), and c.55 C > T (p.R19C) was inherited from her mother(PM2), as shown in Fig. 1. Meanwhile, a heterozygous deletion in *PKHD1* gene exon67 was found. She was diagnosed as ARPKD. Gene variants associated with NVM including *MYH7, ACTC1, TNNT2, MYBPC3, TPM1, TNNI3, TTN*, et al. [7, 8] were not indentified in the patient. After discharge, digoxin 18.75ug twice daily, hydrochlorothiazide 5 mg twice daily, spironolactone 5 mg twice daily, captopril 3 mg three times per day (body weight 7.5 kg) were administered. However, the patient did not take drugs regularly.

**Fig. 2:**
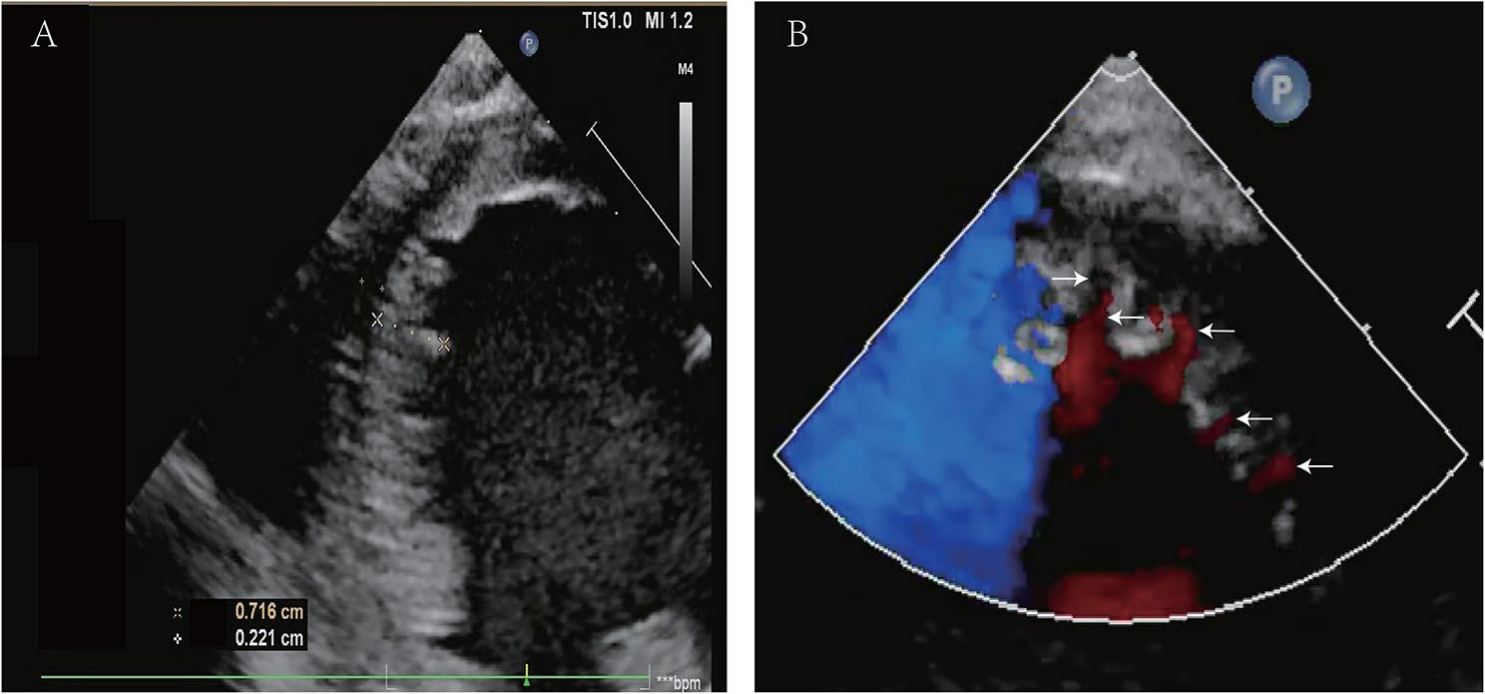
The ultrasound of the patient at 15 month old indicated noncompaction of ventricular myocardium. A: the noncompaction myocardium (× ×) was 0.716 cm and the compaction myocardium (+ +)was 0.221 cm (N/C = 3.27). B: Color doppler imaging showed deep intertrabecular recesses (→) and the appearance of blood flow from the ventricular cavity into the intertrabecular recesses (←)

When the patient was 15 month old, cardiac ultrasound showed prominent trabeculations and deep intertrabecular recesses with the appearance of blood flow from the ventricular cavity into the intertrabecular recesses by color Doppler imaging. The noncompaction myocardium was 0.716 cm and compaction myocardium was 0.221 cm (N/C = 3.27), as shown in Fig. 2, indicating a diagnosis of NVM. An additional movie file shows this in more detail [see Additional file 1]. The left ventricular diastole diameter was 4.3 cm, and the left atrial diastole diameter was 1.8 cm, with an EF of 35% and FS of 17%. Asprin 50 mg per day was prescribed to prevent thromboembolism. At the four-year follow-up, echocardiography showed that EF was 30% and FS was 14%. The cardiac functional grade was a New York Heart Association class II. The liver and kidney functions remained normal. The patient was still being monitored.

## Discussion

Herein, we reported a case of ARPKD accompanied with NVM. The girl was found to have ARPKD at five months old and have normal kidney functions during four years’ follow-up. She was accidently found to have NVM and chronic heart failure.To our knowledge, no cases of ARPKD accompanied with NVM have been previously reported.

Cardiac damage has rarely been reported in patients with ARPKD. Chinali et al. conducted ultrasound examinations on 27 children with ARPKD, of which 17/27 (63%) had a creatinine clearance < 90 mL/min, 3 patients had stage IV chronic kidney disease, 5 patients were on peritoneal dialysis and 19/27 (74%) had hypertension. It was found that compared to 88 normal controls, patients with ARPKD exhibited a higher left ventricular mass index(38.7 ± 12 vs.28.9 ± 4.3 g/[m^2.16^ + 0.09]) and subclinical systolic dysfunction, with a significant decrease in midwall FS and circumferential strain despite normal EF values.Children with ARPKD show significantly impaired cardiac phenotype [9].

ARPKD is a type of renal ciliopathy in which primary cilia play a central role in the pathogenesis [10]. The cilia also play an essential role in regulating the signal cascade required for forming left and right asymmetries in the early stages of cardiac development [11]. Li et al. [12] conducted ultrasound scans on 87,355 chemically mutated C57BL/6J fetal mice. A wide spectrum of congenital heart disease (CHD) phenotypes were recovered, including single pulmonary artery, ventricular septal defect, aortic atresia with ventricular septal defect, heterotaxy, ventricular septal defect, interrupted aortic and so on. 91 recessive CHD mutations in 61 genes were identified by whole exome sequencing, including 34 ciliated genes. Sixteen genes were involved in ciliated cell signaling and ten genes were involved in vesicular transport, including polycystic kidney disease (*PKD*) *1, PKD2, coiled-coil domain containing 151* and dynein assembly factor with WDR repeat domains 1. Confocal imaging of E12.5 Cc2d2a-mutant mouse showed no cilia in the atrioventrcular cushion. Fewer and shorter cilia were observed in other mutant embryo tissue. This suggests that cilia and cilia-induced signal transduction play an essential role in the pathogenesis of CHD.

*PKD*1/2, involved in CHD, are the pathogenic genes for autosomal dominant polycystic kidney disease (ADPKD) [10]. As another kind of PKD, ADPKD was more related to cardiovascular diseases. Of 667 ADPKD patients, 5.8% had idiopathic dilated cardiomyopathy, 2.5% had hypertrophic obstructive cardiomyopathy, and 0.3% had left ventricular noncompaction [13]. There are relationships between the genes of ARPKD and ADPKD. The *PKHD1* gene product FPC co-localizes with the *PKD2* gene product polycystin-2 (PC2) - an intracellular calcium channel expressed in renal epithelial and myocardial cells. After *PKHD1* mutation, a decrease in FPC protein can reduce PC2 and PC2 channel activity [14]. PC2 are expressed in many tissues including vascular smoothmuscle cells and cardiomyocytes. *PKD2* mutations in ADPKD patients could alter the expression of PC2, thus directly impairing intracellular calcium circulation and affecting the function of vascular smooth muscle and cardiomyocytes [15]. Meanwhile, enlargement of cysts in the kidney could lead to renal arteriolar attenuation and ischemia, thus activating renin-angiotensin-aldosterone system. ADPKD could also cause the increase of fibroblast growth factor, then contributing to hypertension and left ventricular hypertrophy [16]. Thus, ADPKD patients may exhibit symptoms such as cardiomyopathy and NVM [13]. The coexistence of ARPKD and NVM may be caused by the alterations of PC1 and PC2 channel activity regulated by FPC. The continuity of NVM with other ciliopathies may indicate that NVM is also a ciliopathy.

In conclusion, the association between ARPKD and cardiac damage remains uncertain. It is unsure if the coexistence of NVM and ARPKD is a coincidence or they are different manifestations of ciliary dysfunction in the heart and kidneys.

### Electronic supplementary material

Below is the link to the electronic supplementary material.

Supplementary Material 1

Supplementary Material 2

## Data Availability

The datasets generated during the current study are available from the corresponding author on reasonable request.

## Abbreviations

ARPKD: Aautosomal recessive polycystic kidney disease
NVM: Noncompaction of ventricular myocardium
ESRD: End-stage renal disease
EF: Ejection fraction
FS: Fraction shortening
PKHD1: polycystic kidney and hepatic disease 1
FPC: Fibrocystin/polyductin
CHD: Congenital heart disease
PKD: Polycystic kidney disease
ADPKD: Autosomal dominant polycystic kidney disease
PC2: Polycystin-2
