# Supplementary material for: A case report of autosomal recessive polycystic kidney disease with noncompaction of ventricular myocardium: coincidence or different manifestations of ciliopathy?
Source: BMC Nephrol. 2024 Jun 25;25:209. doi: 10.1186/s12882-024-03642-7 (PMC11201303; doi:10.1186/s12882-024-03642-7)
Supplement: Supplementary file 2 — Supplementary Material 2 [file 12882_2024_3642_MOESM2_ESM.docx]

**Additional material:**

File name: Additional file 1

Title of data: the ultrasound cardiac movie of the patient
